# Characterization of Migratory Cells From Bioengineered Bovine Cartilage in a 3D Co-culture Model

**DOI:** 10.1177/03635465221113325

**Published:** 2022-08-19

**Authors:** Ming Jia Michael Wu, Corey Sermer, Rita A. Kandel, John S. Theodoropoulos

**Affiliations:** * Lunenfeld-Tanenbaum Research Institute, Toronto, Ontario, Canada; † Institute of Biomaterials and Biomedical Engineering, University of Toronto, Toronto, Ontario, Canada; ‡ Pathology and Laboratory Medicine, Mount Sinai Hospital, Toronto, Ontario, Canada; § Laboratory Medicine and Pathobiology, University of Toronto, Toronto, Ontario, Canada; ¶ Division of Orthopaedic Surgery, Mount Sinai Hospital, Toronto, Ontario, Canada; # Division of Orthopaedic Surgery, University of Toronto, Toronto, Ontario, Canada; Investigation performed at Lunenfeld-Tanenbaum Research Institute, Toronto, Ontario, Canada

**Keywords:** cell migration, bioengineered cartilage, chondrocytes, articular cartilage, integration, platelet-rich plasma

## Abstract

**Background:**

Chondrocyte migration in native cartilage is limited and has been implicated as one of the reasons for the poor integration of native implants. Through use of an in vitro integration model, it has previously been shown that cells from bioengineered cartilage can migrate into the native host cartilage during integration. Platelet-rich plasma (PRP) treatment further enhanced integration of bioengineered cartilage to native cartilage in vitro. However, it is not known how PRP treatment of the bioengineered construct promotes this.

**Hypothesis:**

PRP supports cell migration from bioengineered cartilage and these migratory cells have the ability to accumulate cartilage-like matrix.

**Study Design:**

Controlled laboratory study.

**Methods:**

Osteochondral-like constructs were generated by culturing primary bovine chondrocytes on the top surface of a porous bone substitute biomaterial composed of calcium polyphosphate. After 1 week in culture, the constructs were submerged in PRP and placed adjacent, but 2 mm distant, to a native bovine osteochondral plug in a co-culture model for 2 weeks. Cell migration was monitored using phase-contrast imaging. Cell phenotype was determined by evaluating the gene expression of matrix metalloprotease 13 (MMP-13), Ki67, and cartilage matrix molecules using quantitative polymerase chain reaction. When tissue formed, it was assessed by histology, immunohistochemistry, and quantification of matrix content.

**Results:**

PRP treatment resulted in the formation of a fiber network connecting the bioengineered cartilage and native osteochondral plug. Cells from both the bioengineered cartilage and the native osteochondral tissue migrated onto the PRP fibers and formed a tissue bridge after 2 weeks of culture. Migratory cells on the tissue bridge expressed higher levels of collagen types II and I (*COL2*, *COL1*), *Ki67* and *MMP-13* mRNA compared with nonmigratory cells in the bioengineered cartilage. Ki67 and MMP-13–positive cells were found on the edges of the tissue bridge. The tissue bridge accumulated COL1 and COL2 and aggrecan and contained comparable collagen and glycosaminoglycan content to the bioengineered cartilage matrix. The tissue bridge did not reliably develop in the absence of cells from the native osteochondral plug.

**Conclusion:**

Bioengineered cartilage formed by bovine chondrocytes contains cells that can migrate on PRP fibers and form cartilaginous tissue.

**Clinical Relevance:**

Migratory cells from bioengineered cartilage may promote cartilage integration. Further studies are required to determine the role of migratory cells in integration in vivo.

Articular cartilage is an avascular and paucicellular tissue that covers the ends of long bones. It functions to bear compressive loads and provides low friction articulation of the joint. The extracellular matrix is composed mainly of collagen type II (COL2) and aggrecan (ACAN) that contribute to its tensile strength and compressive resistance, respectively. Mature articular cartilage does not self-repair when damaged, which can result in an increased risk of developing osteoarthritis later in life. This lack of repair is, in part, attributed to the limited ability of chondrocytes to migrate toward sites of injury.

Chondrocytes are surrounded by cartilage matrix that is composed of large proteoglycans compressed within intrafibrillar spaces of collagen networks.^37^ Matrix stiffness, proteoglycan density, and collagen fibril diameter are all factors that have been suggested to hinder the migration of chondrocytes in cartilage.^15,26^ To overcome these barriers and promote migration, studies have used enzymatic digestion to reduce cartilage matrix density.^3,33,42^ However, the use of enzymes risks altering cell phenotype and matrix synthesis.^13^ Isolated chondrocytes have been shown to migrate in response to IGF-1,^4^ PDGF,^23^ FGF,^21^ HMBG-1,^32^ platelet-rich plasma (PRP),^1^ collagen,^36^ and fibronectin^39^ in 2-dimensional monolayer systems. However, chondrocytes dedifferentiate in a 2-dimensional stiff environment and adopt a fibroblastic phenotype, which may alter its migratory behavior.^30^ We have previously shown that cells from bioengineered cartilage migrate into native host cartilage during cartilage integration in vitro.^40^ The inhibition of cell migration prevents cartilage integration, which suggests that cell migration is a crucial mechanism for bioengineered cartilage integration.^20^ Currently, little is known about the phenotype of the migratory cells derived from bioengineered cartilage.

PRP contains high concentrations of bioactive molecules that can stimulate the migration of mesenchymal stem cells (MSCs),^5,29^ chondroprogenitor cells,^18^ and chondrocytes.^1^ We have previously shown that PRP improves the integration of bioengineered cartilage to native cartilage in vitro.^34^ However, it is not known how PRP does this. Thus, the hypothesis of this study is that PRP treatment supports cell migration from bioengineered cartilage and that the migratory cells have the ability to accumulate cartilaginous matrix. This will be demonstrated by showing the effect of PRP treatment on cell migration from bioengineered cartilage in a 3-dimensional (3D) co-culture model. The phenotype of migratory cells and their ability to form cartilage tissue will be characterized. Understanding migratory chondrocytes may help to develop strategies to improve cartilage integration after implantation.

## Methods

### PRP Preparation

Bovine PRP was prepared as described previously.^34^ This was done with research ethics board approval obtained from Ontario Veterinary College, Guelph University (under Mark Hurtig, DVM, MVSc). Briefly, blood from a single animal was drawn into a syringe coated with acid citrate dextrose solution, transferred to a 50-mL tube, and centrifuged for 10 minutes at 200*g*. The plasma layer enriched with platelets was isolated and centrifuged for 10 minutes at 200*g* to remove red and white blood cells to produce PRP. Platelets were counted using a hemocytometer and PRP had a concentration of 1.2 × 10^6^ platelets/µL. PRP was then aliquoted and stored at −80°C until further use.

### Generating Bioengineered Osteochondral-Like Constructs and Native Osteochondral Plugs

**Figure 1.**
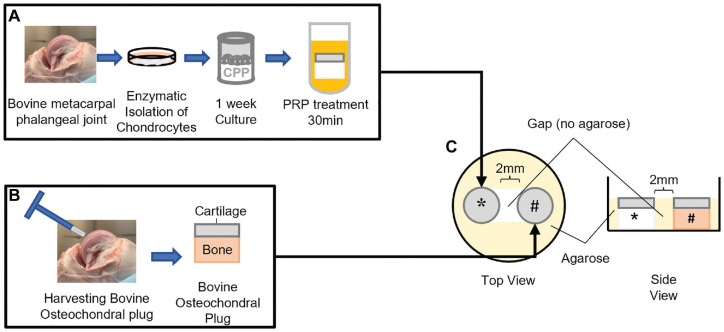
Experiment schematic. (A) Generation of the bioengineered osteochondral-like construct and treatment in 100% platelet-rich plasma (PRP) for 30 minutes before co-culture. (B) Native osteochondral plug harvested with a biopsy punch. (C) 3-dimensional co-culture of bioengineered constructs and native plug immobilized by agarose (yellow) well. *Bioengineered osteochondral-like construct. #Native osteochondral plug.

Porous calcium polyphosphate (CPP) substrates were made as previously described. CPP disks (4-mm diameter, 2-mm height, with an average pore size of 100 µm and porosity of 32%+/- 2.2%) were placed into Tygon tubing (6-mm height, 4-mm inner diameter; No. 3350; Saint-Gobain) to create a well-like structure (Figure 1A) and sterilized by autoclaving.

Full-thickness cartilage was harvested from 1 to 5 bovine metacarpal-phalangeal joints (6-9 months) depending on the experiment. If cells were obtained from more than 1 joint, they were pooled together and considered 1 biological sample. Chondrocytes were isolated by sequential enzymatic digestion of cartilage with 0.25% protease (Sigma-Aldrich) for 45 minutes, followed by 0.1% collagenase A (Roche) for 16 to 18 hours.^34^ The digest solution was filtered sequentially through 100-µm and 40-µm cell strainers.

For selected experiments, superficial zone (SZ) and deep zone (DZ) chondrocytes were differentially isolated as previously described.^6,7^ Briefly, cartilage from the top 10% to 20% (SZ) or bottom 30% to 40% (DZ) of the full-thickness cartilage was harvested with a scalpel. Zone-specific chondrocytes were then isolated via sequential enzymatic digestion as described above.

To generate a bovine osteochondral-like construct, 2 × 10^6^ chondrocytes were seeded onto the top surface of the CPP plug surrounded by Tygon tubing (Saint-Gobain). Cells were cultured in Ham F12 supplemented with 5% fetal bovine serum (FBS) for 2 days and then transferred to Ham F12 supplemented with 20% FBS and ascorbic acid (100 µg/mL final concentration) (Sigma-Aldrich).^34^

Native osteochondral plugs were obtained from bovine metacarpal-phalangeal joints with a 3.5-mm biopsy punch (Smith & Nephew), and excess bone was removed with a scalpel to obtain osteochondral plugs 2 to 3 mm in height.^34,41^ Explants were washed with phosphate-buffered saline (PBS) without Ca^2+^ or Mg^2^ (–/–) and placed in serum-free Ham F12 supplemented with 1% antibiotics overnight under standard culture conditions.

### 3D Co-culture Model

Agarose (4%, 1.25 mL) was pipetted into the wells of a 12-well plate. A dumbbell-shaped agarose well (approximately 10 mm × 4 mm in dimension) was created to ensure that the plugs were held in place and spaced 2 mm apart through the culture duration (Figure 1C).

Native osteochondral tissue was placed in one end of the agarose well (Figure 1C). One-week-old bioengineered constructs were removed from the tubing, soaked in 500 µL of 100% PRP (freeze-thawed once) for 30 minutes, and placed immediately into the other end of the agarose well. Co-cultures were grown in 2 mL of Ham F12 supplemented with 20% FBS and ascorbic acid (100 µg/mL) for up to 2 weeks. Culture medium was changed 3 times a week.

In selected experiments, native osteochondral tissue underwent 3 freeze-thaw cycles (–80°C overnight, followed by 25°C for 30 minutes ×3) before being placed in co-culture or was replaced with a CPP plug (no cells). In other experiments, the cartilage and bone of the osteochondral plug were separated, and the tissues (cartilage or bone) were individually co-cultured with an acellular CPP plug treated with 100% PRP.

### Phase-Contrast Microscopy

Migratory cells were visualized 1 to 2.5 mm above the bottom of the plate in which the PRP fibers were formed by phase-contrast microscopy using a spinning disk confocal microscope (Leica). Phase-contrast images were stitched together using Volocity 3D Image Analysis software (Quorum Technologies).

### Gene Expression

RNA from the co-cultured tissues was isolated after 2 weeks of culture. The tissue bridge, the bioengineered cartilage, and the native cartilage were harvested separately (see Figure 5A) and placed directly into TRIzol reagent (Life Technologies), snap-frozen in liquid nitrogen, and crushed with mortar and pestle.

RNA was extracted using TRIzol reagent according to the manufacturer’s instructions. Total RNA was quantified with a NanoDrop1000 (Thermo Fisher Scientific). RNA (1 µg) was reverse transcribed to cDNA with SuperScript III Reverse Transcriptase (Life Technologies) and amplified by the Mastercycler Thermocycler (Eppendorf). Real-time polymerase chain reaction (RT-PCR) was performed using the Lightcycler 96 RT-PCR system (Roche) with Fast SYBR Green I Master Mix (Life Technologies) and gene-specific primers (Table 1). Relative gene expression was calculated using the Livak method with 18S rRNA as the endogenous control.^19^

**Table 1:** Primers^*a*^

Gene	Primers 5′-3′
*SOX9*	F: GTACCCGCACTTGCACAAC R: GTGGTCCTTCTTGTGCTGC
*ACAN*	F: TGGGACTGAAGTTCTTGGAGA R: GCGAGTTGTCATGGTCTGAA
*COL2*	F: GTGTCAGGGCCAGGATGTC R: GCAGAGGACAGTCCCAGTGT
*COL1*	F: CGGCTCCTGCTCCTCTTAG R: CACACGTCTCGGTCATGGTA
*MMP-13*	F: ATTGATGCCGCCTATGAGCA R: AGGGCTGCGCTGATCTTTTT
*Ki67*	F: GAGACAGCCCAGGACACTTC R: CCTGGTTCTCTGCACCATGT
*PRG4*	F: ATGCCTGAACCGACTCCTAC R: TGCCGA AGCCTTGACTGG
*COL10*	F: CTACAGGCATAAAAGGCCCAC R: GGATGCCTTGCTCTCCTCTCA
*18S rRNA*	F: GTAACCCGTTGAACCCCATT R: CCATCCAATCGGTAGTAGCG

a F, forward; R, reverse.

To confirm enrichment of zone-specific chondrocyte populations, freshly isolated full-thickness (FT), SZ, and DZ chondrocytes were placed into TRIzol reagent. Enrichment of chondrocyte populations was confirmed by differential expression of the zone-specific gene markers proteoglycan 4 (PRG4) (SZ) and collagen type X (COL10) (DZ), as we have done previously.^6^

### Biochemical Analysis

The in vitro–formed cartilage and the tissue bridge were harvested separately and each digested in 40 µg/mL papain (Sigma-Aldrich). The native cartilage removed from the bone was digested in 80 µg/mL papain for 48 hours at 65°C as previously described.^34^

The DNA content of the papain digests was quantified using a fluorometric assay (excitation, 356 nm; emission, 458 nm) and Hoechst 33258 dye (Polysciences) and compared with a standard curve generated using serial dilutions of calf thymus DNA (Sigma-Aldrich) as previously described.^34^

To quantify collagen content, papain digests were acid hydrolyzed for 18 hours at 110°C. Hydroxyproline content was measured using Chloramine-T/Ehrlich’s reagent assay and spectrophotometry (λ = 560 nm) as previously described.^34^ A standard curve was generated with L-hydroxyproline (Sigma-Aldrich).

Sulfated glycosaminoglycan content in the papain digests was quantified using dimethylmethylene blue dye and spectrophotometry (λ = 525 nm) and compared with a standard curve generated using chondroitin sulfate (Sigma-Aldrich) as previously described.^34^

### Histology

After 2 weeks of co-culture, the cartilage tissues were fixed in 10% neutral buffered formalin for 1.5 hours and then placed in 30% sucrose diluted in PBS (–/–) solution overnight at 4°C. Tissues were carefully removed from the CPP and subchondral bone, frozen in Tissue Tek OCT (Sakura Finetek) freezing compound, and sectioned at 7 µm thickness. Tissue sections were stained with hematoxylin and eosin (H&E) or toluidine blue.

### Immunohistochemistry

Tissue sections (7 µm) were pretreated in 2.5 mg/mL pepsin in Tris buffered saline (pH 2.0) for 10 minutes at room temperature for collagen type I (COL1) and COL2 staining, 25 mg/mL hyaluronidase in PBS (–/–) for 30 minutes at 37°C for ACAN staining, or boiled with Dako Target Retrieval solution for 10 minutes for matrix metalloprotease 13 (MMP-13) staining. Sections were blocked with 20% goat serum in 0.1% Triton-X for 1 hour and incubated at 4°C overnight with antibodies reactive with COL1 (1:1000; Abcam 90395), COL2 (1:300; Chemicon MAB8887), ACAN (1:500; Thermo Fisher Scientific AHP0022), or MMP-13 (1:200; GeneTex 59793) suspended in 10% goat serum in 0.1% Triton-X. For Ki67 staining, sections were blocked with 50% SuperBlock (Thermo Fisher Scientific) suspended in 0.1% Triton-X for 1 hour and then incubated at 4°C overnight with Ki67 antibody (1:50; Invitrogen MA5-14520) diluted in the same blocking buffer. The next day, sections were washed in PBS (–/–) and incubated with Alexa 594 goat anti-mouse (1:1000 for COL1; Life Technologies), Alexa 594 donkey anti-rabbit (1:500 for MMP-13, 1:1000 for Ki67; Life Technologies), or Alexa 488 goat anti-mouse (1:1000 for COL2 and ACAN; Life Technologies) at room temperature for 1 hour. Sections were washed with PBS (–/–), nuclei stained with DAPI (Thermo Fisher Scientific) for 10 minutes, and coverslipped with Permafluor Mounting Agent (Thermo Fisher Scientific). Tissues were visualized using an Optigrid fluorescent microscope (Leica).

### Ki67 Cell Counting

Ki67-positive cells were counted in tissues immunohistochemically stained with antibody reactive to Ki67. Each biological experiment contained 3 replicate tissues, and 2 sections were used from each replicate tissue. In each tissue section, 5 standardized regions of the tissue bridge and the bioengineered cartilage were imaged at ×20 magnification with an Optigrid fluorescent microscope (Leica). Ki67-positive cells were counted using ImageJ software. A binary threshold filter was applied to each image and all Ki67-positive signals that overlap with nuclei were considered Ki67-positive cells and expressed as a percentage of total number of nuclei.

### Statistical Analysis

All experiments were repeated 3 to 4 times using separate biological samples, and each condition was done in triplicate unless stated otherwise. For the gene expression studies, 4 to 5 individual bridge tissues were pooled to represent 1 technical replicate. For biochemical analysis, 3 individual tissues (bioengineered cartilage, tissue bridge, or native cartilage) were combined for 1 technical replicate. RNA expression and biochemical analysis were displayed as scatterplots, with each point representing the average value of an independent experiment. The bars represent standard deviation. The Student *t* test or 1-way ANOVA with Tukey’s post hoc analysis was used to detect differences between 2 or more than 2 groups, respectively. Significance was assigned at *P* < .05. Values that were outside of Q1–1.5IQR or Q3+1.5IQR were considered outliers and excluded to prevent outliers from inappropriately influencing the significance of the data.^28^

## Results

### Cells Migrate Onto PRP Fiber Network

**Figure 2.**
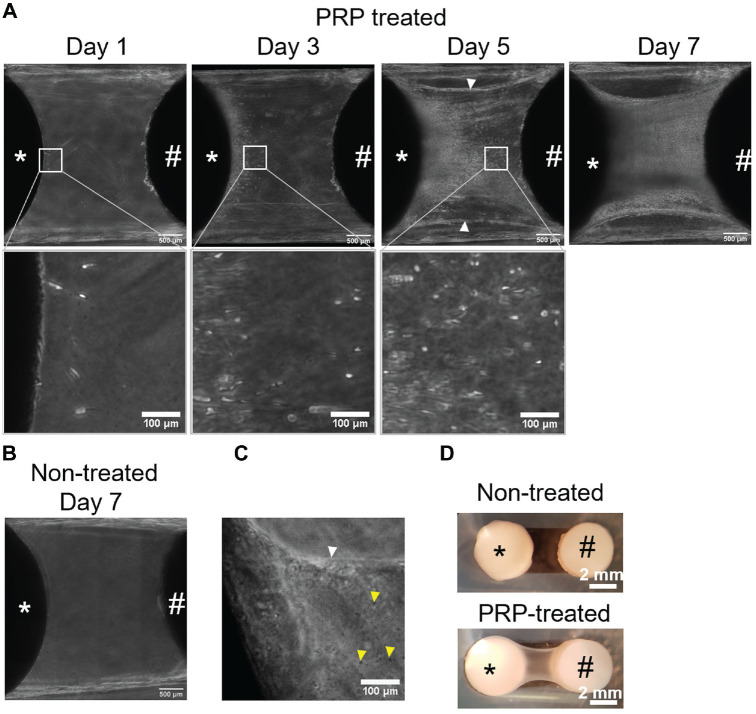
Chondrocytes migrate onto platelet-rich plasma (PRP) fiber network. (A) Phase-contrast images of co-culture at days 1, 3, 5, and 7. On day 7, cells can be seen migrating across the entire fiber network. The white arrowhead points to the PRP fiber network. (B) Phase-contrast image of non–PRP treated construct at day 7 of co-culture showing no fiber formation and no cell migration. (C) Phase-contrast image of PRP fibers (white arrowhead) present 1 day after PRP soaking. Structures that resemble platelets (yellow arrowheads) can be seen within the fibers. (D) Macroscopic appearance of co-cultured constructs and the bridge that forms between them by 14 days. *Bioengineered construct. #Native osteochondral explant.

On day 1 of co-culture, a fiber network with entrapped structures that resemble platelets could be visualized (Figure 2, A and C). These PRP fibers connected the bioengineered construct and native osteochondral plug. Cells from the bioengineered cartilage began to migrate onto the PRP fibers on day 1 of co-culture (Figure 2A); they were between 1 and 2 mm above the bottom of the plate. These migratory cells had either spherical or elongated morphology (Figure 2A). By days 5 to 7, cells had migrated across the PRP fibers to reach the native cartilage 2 mm away. By 2 weeks of co-culture, migratory cells had formed a “bridge” connecting the bioengineered and native cartilages (Figure 2, A and D). The fibers did not form without PRP treatment, and there was no bridge as the cells did not have a scaffold onto which to migrate (Figure 2, B and D). Cells from the native osteochondral plug also migrated onto the PRP fibers as early as day 3 of the co-culture period in 6 of 8 native osteochondral plugs (N = 3, n = 2-3; N, biological samples; n, technical replicates).

**Figure 3.**
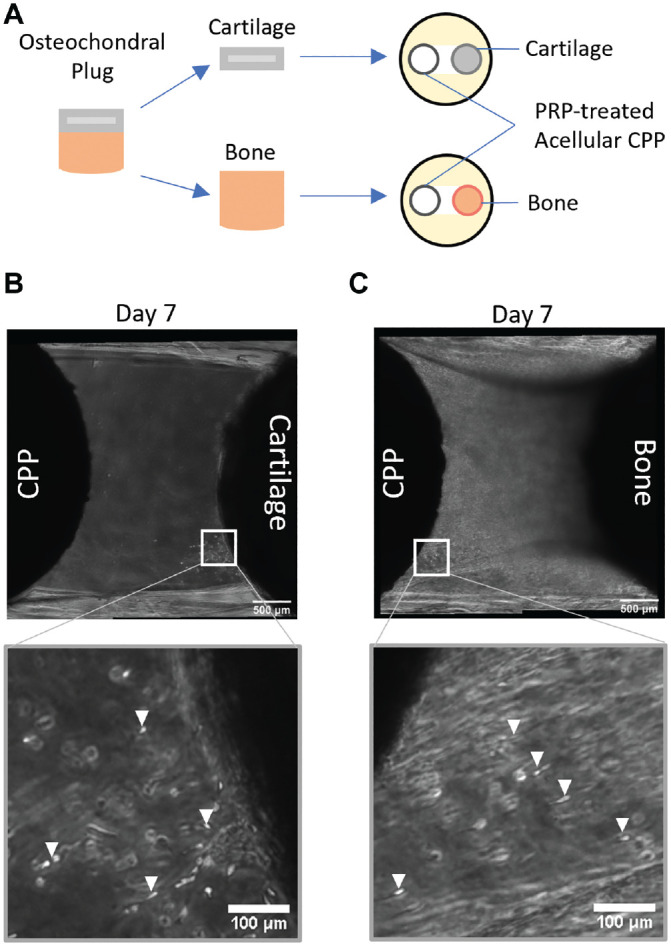
Limited cell migration from cartilage disk and abundant cell migration from native bone tissue. (A) Schematic of experimental design showing separation of cartilage and bone from the osteochondral plug. Phase-contrast images of platelet-rich plasma (PRP)–treated acellular bone substitute biomaterial (calcium polyphosphate [CPP]) co-cultured with either (B) native cartilage disk or (C) bone plug on day 7. The white box indicates areas of higher magnification; the white arrowheads indicate migrating cells.

To determine the origin of the cells from the native tissue, the native osteochondral plug was separated into a cartilage disk and a bone plug (no cartilage), and each was individually co-cultured with a PRP-treated acellular CPP disk (Figure 3A). An acellular CPP disk was used so that if cells were seen migrating, they had to be from the native plugs. A limited number of cells migrated out of 4 of 9 native cartilage disks (N = 3, n = 3) by day 7 of co-culture (Figure 3B). In comparison, cells from all the bone plugs had migrated across and populated the PRP fibers by day 7 (N = 3, n = 3) (Figure 3C).

**Figure 4.**
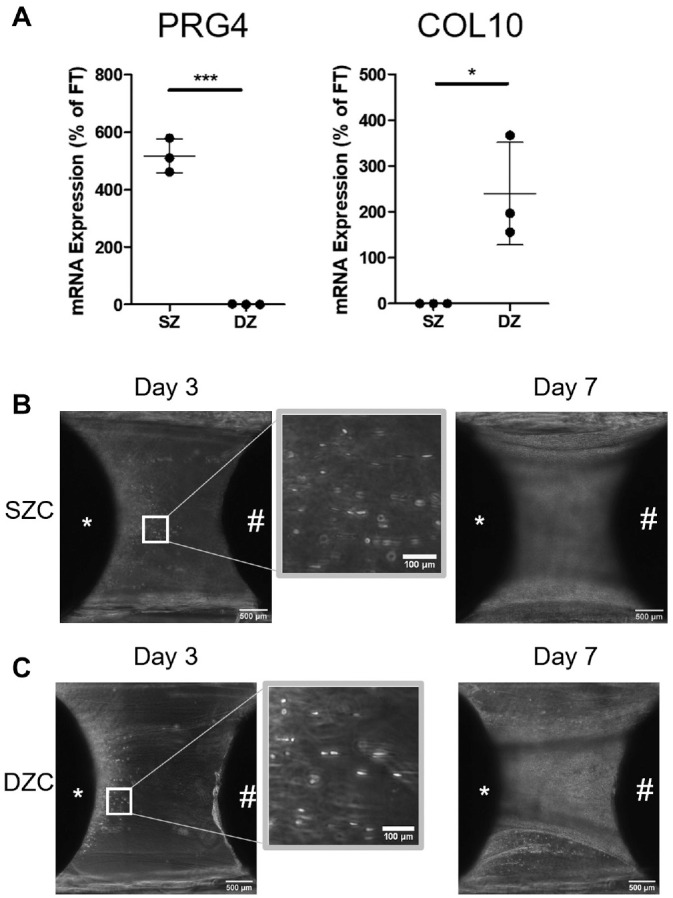
Superficial (SZ) and deep (DZ) zone chondrocytes (SZC and DZC) migrate onto platelet-rich plasma (PRP) fiber. (A) Enrichment for SZC and DZC after zonal cell isolation was demonstrated by differential gene expression of *PRG4* and *COL10*. N = 3; *P ≤ .05; ***P ≤ .0005. Phase-contrast images of migratory cells from bioengineered cartilaginous tissue formed by either (B) SZCs or (C) DZCs at 3 days or 7 days after PRP treatment. *Bioengineered construct. #Native osteochondral explant. The white box indicates areas of higher magnification. COL, collagen; PRG, proteoglycan.

Bioengineered tissues formed by SZ or DZ chondrocytes (SZC or DZC, respectively) were generated to determine if the migratory cells derive from a specific zone of cartilage. SZCs and DZCs were isolated from approximately the top 20% and bottom 30% of full-thickness cartilage. Growing zone-specific cells in 3D has been shown to support the maintenance of their phenotype.^38,44^ Enrichment of SZCs or DZCs was confirmed by differential expression of lubricin (PRG4) and COL10 (Figure 4A). On day 3 of co-culture, cells from either SZC- or DZC-developed bioengineered cartilage could be seen migrating across the PRP fibers (Figure 4, B and C). By day 7, cells from both SZC- and DZC-developed bioengineered cartilage had migrated across the fibers and reached the native tissue, similar to full-thickness cells (Figure 4, B and C).

### Gene Expression of Migratory Cells

**Figure 5.**
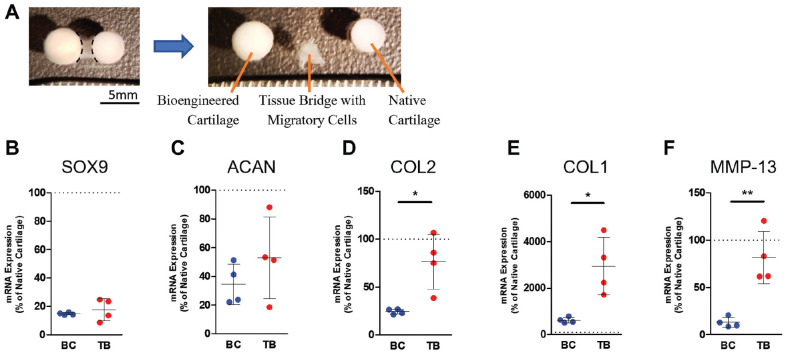
Gene expression of migratory chondrocytes after 14 days of co-culture. (A) Bioengineered cartilage (BC) and the tissue bridge (TB) were harvested separately for RNA isolation and gene expression analysis. The dashed lines show the region where the tissue bridge was separated. mRNA levels of (B) *SOX9*, (C) *ACAN*, (D) *COL2*, (E) *COL1*, and (F) *MMP-13* of cells in tissue bridges were compared with those of BC chondrocytes. mRNA expression was expressed as the percentage of freshly harvested (not cultured) native cartilage. The data are shown as a scatterplot, with each point representing the mean value of 1 independent experiment. The bars indicate ± SD. The dotted line indicates the native cartilage level of expression. *P ≤ .05; **P ≤ .005 between TB and BC. N = 4. ACAN, aggrecan; COL, collagen; MMP, matrix metalloprotease; SOX, SRY-Box Transcription Factor.

The tissue bridge that contained the migratory cells was separated from the bioengineered cartilage and the native cartilage, and RNA from each tissue was isolated (Figure 5A). Migratory cells and bioengineered cartilage chondrocytes expressed similar levels of chondrogenic genes, *SOX9* and *ACAN* (Figure 5, B and C). However, the migratory cells expressed significantly higher levels of *COL2*, a cartilage-specific matrix gene, *COL1*, and *MMP-13* compared with bioengineered cartilage chondrocytes (Figure 5, D-F).

### Biochemical Analysis of Tissue Bridge

**Figure 6.**
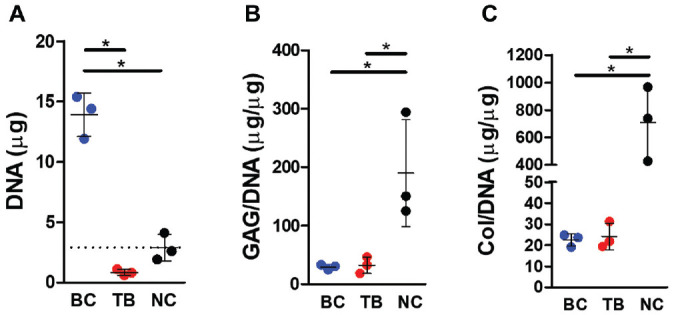
Biochemical analysis of tissues. (A) DNA content, (B) glycosaminoglycan (GAG) content, and (C) collagen (Col) content of bioengineered cartilage (BC), tissue bridge (TB), and native cartilage (NC). N = 3 independent biological replicates; *P ≤ .05.

DNA, glycosaminoglycan (GAG), and collagen contents of the tissue bridge, bioengineered cartilage, and native cartilage were compared. The tissue bridge and the bioengineered cartilage contained similar GAG and collagen contents when normalized to DNA (Figure 6, B and C). Both bioengineered cartilage and the tissue bridge had significantly lower GAG and collagen accumulation/cells compared with native cartilage.

### Histological and Immunohistochemical Analysis of Tissue Formed by Migratory Cells

**Figure 7.**
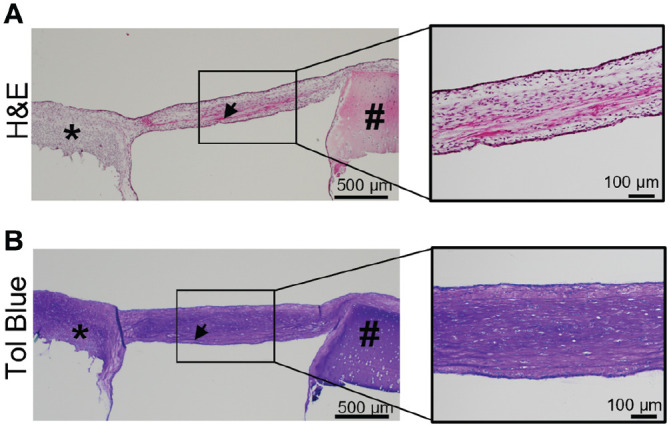
Histological appearance of the tissue bridge formed by migratory cells. (A) Hematoxylin and eosin– and (B) toluidine blue–stained tissue sections. The box indicates the location of higher-magnification insets. *Bioengineered cartilage. #Native cartilage; the black arrow indicates entrapped fibers. N = 3 (1 technical replicate).

**Figure 8.**
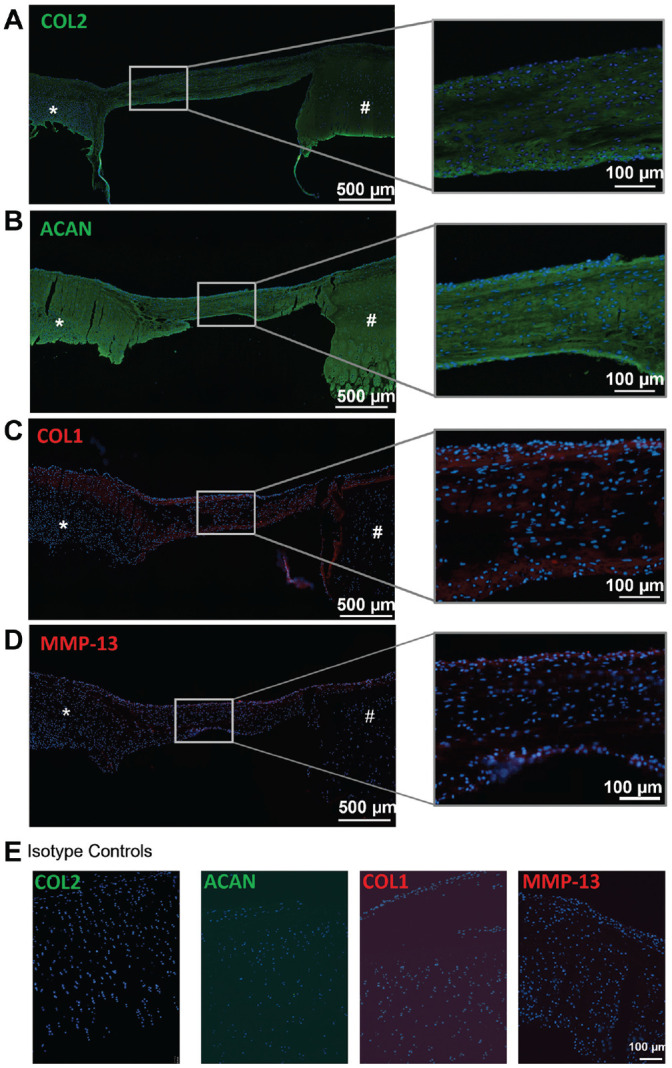
Composition of tissue bridge formed by migratory cells. Immunohistochemical staining of tissue sections using antibodies reactive with (A) COL2, (B) ACAN, (C) COL1, or (D) MMP-13. (E) Isotype negative controls for COL2, ACAN, COL1, and MMP-13. *Bioengineered cartilage. #Native cartilage. The white box indicates the site of the magnified image of the tissue bridge. N = 3 biological replicates, (1 technical replicate). ACAN, aggrecan; COL, collagen; MMP, matrix metalloprotease.

Histological analysis showed that migratory cells accumulated cartilage-like tissue that incorporated the PRP fibers and generated a tissue bridge that connects the bioengineered and native cartilage by day 14 (Figure 7A). The tissue bridge was rich in proteoglycans as determined by toluidine blue staining (Figure 7B). Immunohistochemical staining showed that the extracellular matrix of the tissue bridge contained COL2 (Figure 8A) and ACAN (Figure 8B). COL1 and MMP-13 were also detected in the superficial and inferior aspects of the tissue (Figure 8, C and D).

### Cells From Native Osteochondral Plug Enhance Matrix Accumulation by Migratory Cells From Bioengineered Cartilage

**Figure 9.**
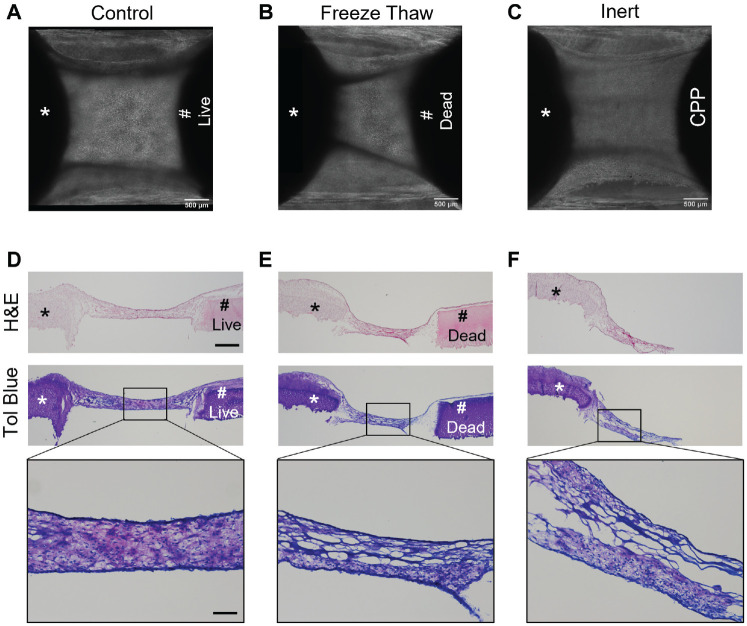
Native osteochondral plug enhances matrix formation by migratory cells from bioengineered cartilage. (A-C) Phase-contrast images. (D-F) Hematoxylin and eosin– and corresponding toluidine blue–stained tissues of co-cultures of a bioengineered construct with (A, D) viable osteochondral plug, (B, E) osteochondral plug (freeze-thaw), and (C, F) acellular calcium polyphosphate (CPP). *Bioengineered cartilage. #Native osteochondral plug. The box indicates the region of tissue imaged at higher magnification. Scale bar = 500 µm or 100 µm in magnified images. N = 3 biological replicates (2 technical replicates).

To determine if cells from the native osteochondral plug affect migration and matrix accumulation on the PRP fibers, the osteochondral plug underwent 3 freeze-thaw cycles to kill the cells and then were placed in co-culture. In separate experiments, the bioengineered construct was co-cultured with acellular CPP (no tissue). In the standard co-culture condition, cells from both the bioengineered and native osteochondral plug migrated onto the fibers and formed a cartilaginous tissue bridge in all experiments (Figure 9, A and D). In co-cultures with freeze-thaw treated osteochondral plugs or acellular CPPs, only cells from bioengineered cartilage migrated onto the fibers over time (Figure 9, B and C). When cells were present on the fibers, the cells appeared to accumulate less extracellular matrix as determined by toluidine blue staining in 2 of 6 (N = 3, n = 2) samples compared with the standard condition in which all co-cultures formed cartilaginous tissue (Figure 9, D-F).

### Migratory Cells Express Higher Levels of Ki67 Than Chondrocytes in the Bioengineered Cartilage

**Figure 10.**
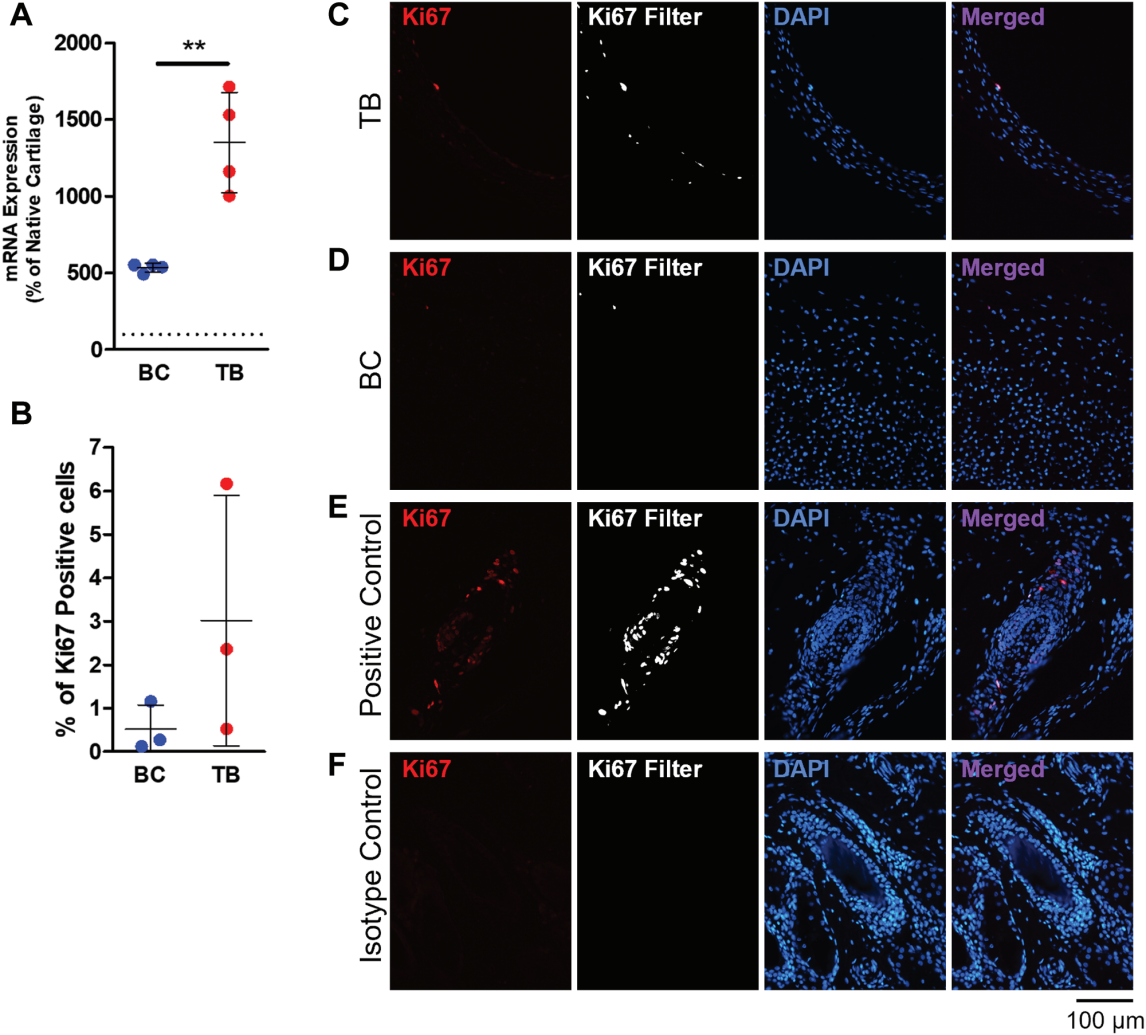
Ki67 gene and protein expression after 2 weeks of co-culture. (A) Relative gene expression of *Ki67* mRNA in the bioengineered cartilage (BC) and tissue bridge (TB). Gene expression data presented as a percentage of freshly harvested (not cultured) native cartilage. (B) Ki67-positive cells in the BC and TB were counted in immunostained tissues and expressed as a percentage of the total number of nuclei. (A, B) The data are shown as a scatterplot, with each point representing the mean value of 1 independent experiment. The bars indicate ± SD. **P ≤ .005 between TB and BC. N = 3-4. Immunohistochemical staining with antibody reactive to Ki67 of the (C) TB, (D) BC, and (E) positive control (bovine hair follicles). (F) An isotype negative control antibody was used to detect nonspecific binding.

Two-week co-cultured tissues were harvested to evaluate cell proliferation by determining Ki67 gene and protein expression. RT-PCR analysis demonstrated a significantly higher level of *Ki67* mRNA expression in the migratory cells in the tissue bridge compared with the nonmigratory cells in the bioengineered cartilage (*P* = .0025) (Figure 10A). Immunohistochemical staining showed that there may be a higher percentage of Ki67-positive cells on the tissue bridge compared with the bioengineered cartilage, but the difference was not statistically significant when quantified (*P* = .2527) (Figure 10, B-E). Ki67-positive cells were present mainly at the edges of the tissue bridge and on the superficial aspect of the bioengineered cartilage that is contiguous with the tissue bridge (Figure 10, A and B).

## Discussion

In summary, a 3D co-culture model composed of a bioengineered osteochondral-like construct formed using a bone substitute biomaterial treated with PRP and a native bovine osteochondral plug was developed. PRP treatment resulted in the formation of a fiber network that connected the bioengineered cartilage and the native tissue that were 2 mm apart. Cells from bioengineered cartilage and native bone migrate onto PRP fibers to accumulate cartilaginous tissue composed of COL2, ACAN, and COL1. Cells on the superior and inferior aspect of the tissue bridge express Ki67 and MMP-13. Migratory cells from bioengineered cartilage do not appear to originate from a specific zone of cartilage as cells migrated from tissues formed by either SZ or DZ chondrocytes. Compared with chondrocytes in the bioengineered cartilage, migratory cells expressed similar levels of chondrogenic genes, *ACAN* and *SOX9*, and higher levels of *COL2*, *COL1*, *Ki67*, and *MMP-13*. Additionally, cells from the native bone appear to enhance matrix accumulation by migratory cells.

In this model, we hypothesized that the PRP fibers are fibrin formed from the residual PRP on the bioengineered construct after exposure to the calcium in the culture medium, as has been described by others.^41,43^ The PRP fibers formed between the construct and the plug likely because no agarose is present in this region. The PRP fibers acted as a preliminary scaffold onto which cells migrate and accumulate matrix. PRP scaffolds have been shown to support chondrogenic differentiation of chondroprogenitors,^43^ MSCs,^47^ and chondrocytes.^46^ The biocompatibility, lack of immunogenicity, and high concentration of autologous growth factors make PRP a good biological scaffold for chondrocytes. We have previously demonstrated that PRP treatment of bioengineered constructs enhanced the integration of bioengineered cartilage to native cartilage in vitro.^34^ It is possible that the formation of PRP fibers and the migration of cells in the gaps between tissues may be the way by which PRP enhances integration.

Other studies have suggested that cell migration from native cartilage tissue is limited and cannot reliably contribute to cartilage integration.^24,33,40,48^ This is in keeping with our observations, as only a limited number of cells were seen to migrate out of native cartilage. However, we observed that cells in bioengineered cartilage, which has less extracellular matrix, are able to migrate out of the tissue in all samples. This begins as early as day 1 of co-culture, and the cells can migrate across the 2-mm gap by days 5 to 7. The migratory cells observed in this study are likely the same cells that we previously reported to migrate into native host cartilage during integration.^40^ The migratory cells do not appear to be chondrocytes from a specific zone as both SZC and DZC are able to migrate across the PRP fibers. This is consistent with a study showing that isolated SZC and DZC have comparable migratory abilities in a Transwell assay.^12^ Interestingly, we observed cells migrating out of the bone of the osteochondral plugs. Although not characterized, these are likely to be MSCs from the bone marrow, which have also been shown to migrate in response to PRP.^29^

Cell phenotype and matrix deposition play important roles in cartilage-cartilage integration.^9,24,27^ In this study, migratory cells on the PRP fibers appear to maintain a chondrogenic phenotype, as demonstrated by the similar gene expression of *SOX9* and *ACAN*, and increased expression of *COL2* relative to the nonmigratory chondrocytes in bioengineered cartilage. The migratory cells also retained the ability to accumulate cartilaginous matrix composed of COL2, COL1, and ACAN, and contained a similar collagen and GAG content compared with the bioengineered cartilage.

Although gene and protein expression of COL1 was unexpected, this may be attributed to either the presence of admixed cells from the native bone or the dedifferentiation of proliferating cells on the fibers. Interestingly, cells from the native bone plug appeared to enhance matrix accumulation on the fibers. Without bone-derived cells, migratory cells from bioengineered cartilage alone were unable to form a tissue bridge consistently. The reason for this is unknown, but it has been shown by others that co-culture of MSCs with chondrocytes resulted in enhanced matrix accumulation.^8,22^ The presence of both cell types may be needed to provide sufficient cell density to give rise to cartilage-like tissue.^35^

Migratory cells on the tissue bridge appeared to have the capacity to proliferate, as demonstrated by the significantly higher level of *Ki67* mRNA compared with the bioengineered cartilage, as well as the presence of Ki67-positive cells in the bridge tissue. The high variance in the number of Ki67-positive cells and mRNA observed in the tissue bridge may be attributed to the different states of differentiation of the cells as they migrate and accumulate tissue. Interestingly, the proliferative cells were located primarily on the edges of the bridge tissue and the superficial aspect of the bioengineered cartilage that is contiguous with the tissue bridge. This suggests that the cells that migrate from the tissues may also proliferate.

Similar to Ki67, MMP-13 is expressed by cells at the edges of the bridge tissue. MMP-13 is highly expressed in migrating chondroprogenitors after cartilage injury to promote migration by degrading the surrounding matrix.^2,26,32^ Interestingly, MMP-13 may also play a role in chondrogenesis as it is expressed during MSC chondrogenesis and by chondroprogenitors.^31,32^ However, the role of MMP-13 in our system has not been elucidated and this requires further investigation.

Recently, studies have isolated chondroprogenitor populations based on their ability to migrate out of cartilage explants.^10,32^ This raises the question of whether the migratory cells observed in this study are also chondroprogenitors.^16,32^ Chondroprogenitors are resident chondrocyte precursors that play a role in cartilage repair and have been shown to migrate in response to cartilage injury and in osteoarthritis.^16,32^ Like MSCs, chondroprogenitors are highly clonogenic and undergo trilineage differentiation, but they are more committed to the chondrogenic lineage.^14,16,32^ Therefore, they are a promising source of cells for cartilage tissue engineering. Seol et al^32^ and Koelling et al^16^ showed that their chondroprogenitor cell underexpresses chondrogenic genes such as COL2 and ACAN and overexpresses COL1- and RUNX 2–relative chondrocytes, but they can undergo chondrogenic differentiation in response to TGFβ3, BMP-6, and PRP.^17,45^ In addition, chondroprogenitors have significant upregulation of proliferative and migratory genes, including MMP-13 expression. Migratory cells in this study share similarities with chondroprogenitors based on their proliferative, chondrogenic, and migratory phenotype. It is possible that the higher levels of chondrogenic gene expression observed in our migratory cells are a result of chondrogenic differentiation induced by the PRP treatment or the co-culture setting. Further investigation is needed to determine if these migratory cells are truly chondroprogenitors.

While the 3D co-culture model made it possible to study the migratory cells, it has several limitations. First, the 2-mm gap between bioengineered construct and osteochondral plug is a distance that is unlikely to occur for cartilage implants. This distance was selected to visualize cell migration and to characterize the tissue formed by migratory cells. Second, the use of PRP from a single animal is a limitation of this study. Platelet count and growth factor concentration can vary between biological samples,^11^ which may influence the chondrogenic differentiation of cells.^17^ Third, we did not label the bioengineered cells, so we were unable to determine the relative contribution of cells from the bioengineered cartilage and native plug to the matrix accumulation on the PRP fibers. In addition, the cells migrating out of the osteochondral plug were observed to come mainly from the bone, but the cell type was not determined. We hypothesize that these cells are MSCs, which can also explain the accumulation of COL1 in the tissue bridge, as they are known to produce this collagen type.^25^ Performing scRNA-seq analysis in a future study can help to identify the contribution of cells from the different tissue types and elucidate the mechanism that allows these cells to migrate and accumulate extracellular matrix.

## Conclusion

This study demonstrated that cells from both bioengineered cartilage and native bovine bone migrate on PRP fibers in a 3D co-culture model. These cells have a migratory, proliferative, and chondrogenic phenotype. Together, they accumulate cartilaginous tissue containing COL2, ACAN, and some COL1. Further studies are necessary to determine if the PRP fibers and these migratory cells contribute to the integration of bioengineered cartilage to native cartilage in vivo.
